# Pheno- and genotypic characterization and identification of novel subtypes of Peste des Petits Ruminants virus in domestic and captive wild goats in Northern Iraq

**DOI:** 10.1186/s12866-021-02372-2

**Published:** 2021-12-07

**Authors:** Faisal Polis Khoran, Elham Potros Candlan, Abdulwahed Ahmed Hassan, Fanar A. Isihak, Amir Abdulmawjood, Izhar U. H. Khan

**Affiliations:** 1 Directorate of Central Veterinary Laboratory (DCVL), Erbil, Kurdistan Region Iraq; 2 Department of Veterinary Public Health (DVPH), College of Veterinary Medicine, University of Mosul, Mosul, Iraq; 3 Agriculture and Agri-Food Canada, Ottawa Research and Development Centre, Ottawa, ON K1A 0C6 Canada; 4 Department of Veterinary Microbiology, College of Veterinary Medicine, University of Mosul, Mosul, Iraq; 5 Institute of Food Quality and Food Safety, University of Veterinary Medicine Hannover, Bünteweg 17, D-30559 Hannover, Germany

**Keywords:** Peste des Petits ruminants, Domestic goat, Captive wild goat, Nucleoprotein (N) gene, Re-emergence

## Abstract

**Background:**

Peste des Petits Ruminants (PPR) is an acute or peracute contagious transboundary viral disease that mainly affects caprine and ovine and causes significant economic impact in developing countries. After two PPR virus outbreaks in 2011 and 2014, an investigation, from August 2015 to September 2016, was carried out in Northern Iraq when an increased morbidity and mortality rates were reported in the domestic and captive wild goats. In the present study, ten domestic goat farms and seven captive wild goat herds located in seven geographical areas of Northern Iraq were clinically, pathologically, serologically and genotypically characterized to determine the prevalence and potential cause of PPR virus outbreak.

**Results:**

The outbreak occurred with rate of morbidity (26.1%) and mortality (11.1%) in domestic goat farms as compared to captive wild goat herds where relatively high mortality (42.9%) and low morbidity (10.9%) rates were recorded. Based on the clinical symptoms (mucopurulent nasal discharges, ulceration and erosion of oral mucosa, profuse watery diarrhea) and necropsy (hemorrhage and congestion on mucous membranes of the colon and rectum with zebra stripes lesions) results, overall, the serological test findings revealed a high frequency (47.9%) of positive samples for anti-PPRV nucleoprotein antibodies. Furthermore, the nucleoprotein (N) gene was detected in 63.2 and 89.1% of samples using conventional and reverse transcription real-time quantitative PCR assays. A phylogenetic analysis of N gene amino acid sequences clustered with the reference strain revealed lineage IV similar to the strains isolated in 2011 and 2014, respectively. However, two sub-types of lineage IV (I and II), significantly distinct from the previous strains, were also observed.

**Conclusion:**

The phylogenetic analysis suggests that movements of goats are possible cause and one of the important factors responsible for the spread of virus across the region. The study results would help in improving farm management practices by establishing a PPR virus eradication program using regular monitoring and vaccination program to control and mitigate the risk of re-emergence of PPR virus infection in domestic and captive wild goats in Iraq.

**Supplementary Information:**

The online version contains supplementary material available at 10.1186/s12866-021-02372-2.

## Abstract (article highlights)

1. Assessment and detection of source of re-emergence of Peste des Petits Ruminants virus in goats.

2. High rate of mortality than morbidity in domestic and captive wild goat herds.

3. Identification of novel sub-types (I&II) of lineage IV.

4. Regular monitoring and strategies for improving on-site control and trade regulations enables mitigating risk of outbreaks.

**Supplementary Information:**

The online version contains supplementary material available at 10.1186/s12866-021-02372-2.

## Background

The Peste des Petits Ruminants (PPR) is a single stranded non-segmented negative-sense RNA virus that belongs to the genus *Morbillivirus*, family *Paramyxoviridae* and order *Mononegavirales* [1]. The virus can generate eight proteins encoding six structural (N, P, M, F, H and L) and two nonstructural accessory (C and V) proteins [2, 3]. The PPR virus has been classified into four lineages (I, II III and IV) based on the partial fusion (F) protein gene, the nucleoprotein (N) gene or the haemagglutinin (H) glycoprotein gene. Geographically, lineages I, II and III are mostly prevalent in Africa; while lineage IV has been found in almost all Asian and several African countries [3].

The PPR virus is an endemic and highly contagious viral disease of small ruminants that has caused significant economic losses mostly affecting goat farms with high morbidity (80–90%) and mortality (50–80%) rates [4, 5]. Transmission of PPR infection in farm animals is commonly caused by inhalation and direct contact with contaminated ocular, nasal, and oral secretions as well as feces of infected animals. In addition, bedding, feed and water troughs can also serve as a source of disease transmission [4, 6].

For the diagnosis of PPR infections, various serological and molecular methods including agar gel immunodiffusion, counter-immunoelectrophoresis, competitive enzyme-linked immuno-sorbent (c-ELISA), blocking ELISA (b-ELISA), reverse transcription quantitative PCR (RT-qPCR) and reverse transcription loop-mediated isothermal amplification (RT-LAMP) have been widely applied [7–9].

The PPR disease is, currently, endemic in Iraq where most of the outbreaks in sheep as well as domestic and wild goat have been reported in the Northern region. In 1997, the disease was first time studied in sheep herds in the central and northern regions of Iraq with a sero-prevalence rate of 21.6 and 30.9%, respectively [10]. However, in 1998, the first outbreak was reported in Nineveh Governorate followed by another outbreak in 2000 where high morbidity (70%) and low mortality (8%) rates in lambs less than 4 months of age were reported in two sheep herds near Baghdad Governorate [11, 12]. Later, the rate of sero-prevalence (31.2%) of PPR virus, with no clinical symptoms, was recorded in domestic goats and sheep in 14 Governorates across the country [13, 14]. Another PPR outbreak in wild goats (bezoar ibex, *Capra aegagrus*), between August 2010 and February 2011, was reported in Erbil Governorate of Northern Iraq; however, in 2012, the genetic lineage IV was detected for the first time in wild goats [15]. The second wave lasted until October 2011 that also caused significantly high rate of mortality in wild goats (*Capra aegagrus*) [16]. Later, another outbreak, genetically associated with lineage IV, occurred on sheep farms in Al-Sulaimaniyah Governorate located in the northeastern part of Iraq [17]. Recently, a PPR outbreak, between August 2015 and September 2016, in ten domestic goat farms and seven captive wild goat herds was reported in different geographical regions of Erbil Governorate of Northern Iraq.

With this re-emergence of PPR infection, the present study was carried out to: i) investigate the rate of prevalence of PPR virus in domestic and captive wild goats; ii) identify the source of infection by genotypic characterization of PPR strains and compare with 2011 and 2014 viral strains data; and iii) investigate the introduction of novel strains due to unrestricted movement of the animals across the border. The study results would aid in developing control strategies to prevent the spread of future PPR virus outbreaks in the region.

## Results

### Clinical observations

**Fig. 1:**
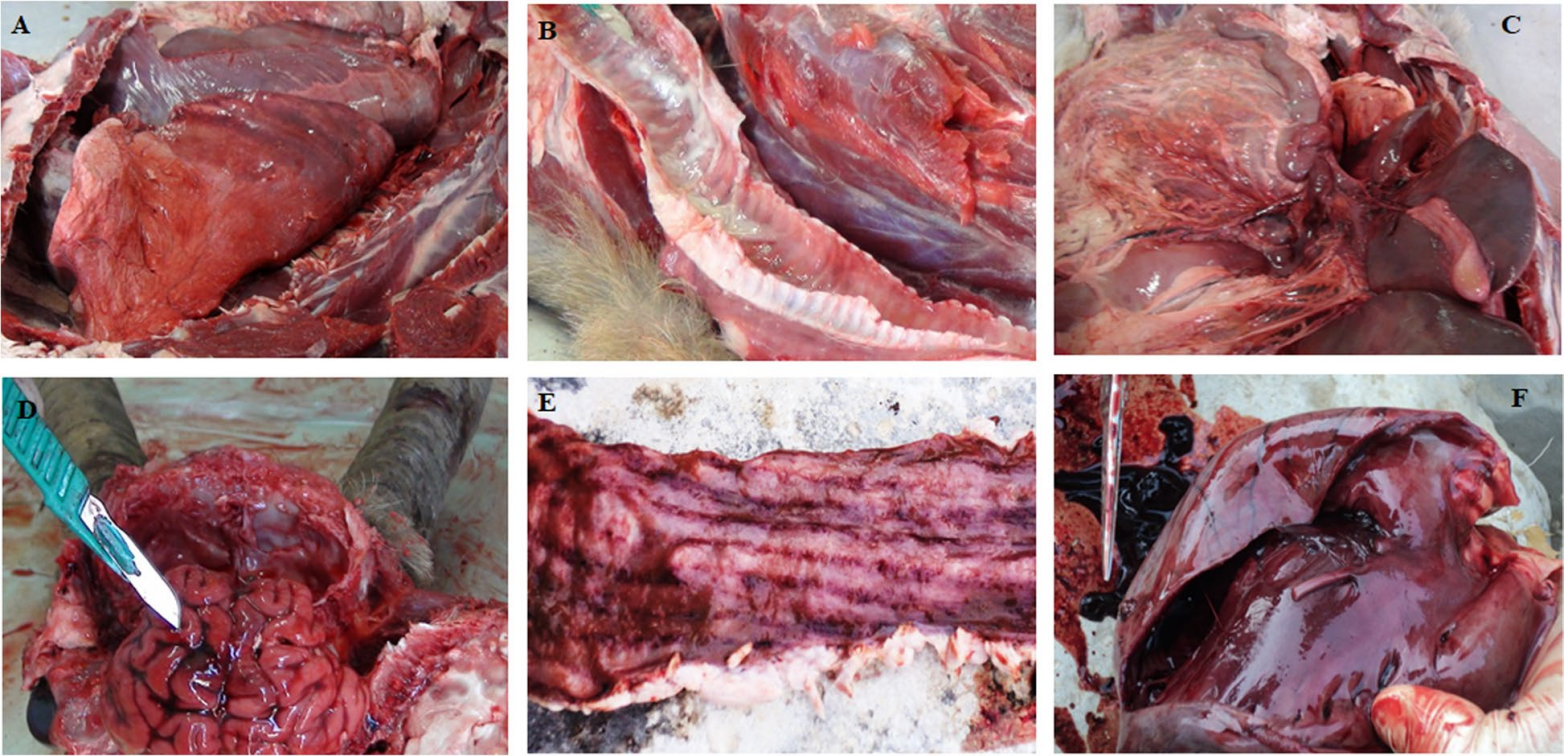
Postmortem lesions of PPR virus infection observed in domestic and captive wild goats. Panels **A-F**: **A**&**B**: Hemorrhages and congestion of the lungs and trachea contained foamy fluid; **C**: Hemorrhages and congestion on the abdominal mesenteric tissue; **D**: Hemorrhage and congestion of the brain; **E**: Zebra stripes in the mucous membranes of colon and rectum; **F**: Hemorrhage and congestion of liver

Infected animals showed clinical (peracute, acute or subacute) symptoms ranging from mild to severe and sudden death. The non-specific clinical symptoms including high fever (up to 41.7 °C), moderate to profuse watery diarrhea, emaciation, loss of appetite (anorexia), lameness and paralysis of legs were observed. The specific PPR infection signs were mucopurulent and bloody nasal discharge and putrid breath odor, ulceration and erosion of oral mucosa inside of the lower lips and free portion of the tongue (erosive stomatitis), congestion of conjunctiva (conjunctivitis), respiratory distress and depression. In addition, several cases of abortion were recorded (Table 1; Fig. 1A-F).

**Table 1:** Geographical, clinical and vaccination data of domestic goat farms (*n* = 10) and captive wild goat herds (*n* = 7) investigated in this study

Animal	Management type	Geographical location	Herd or Farm code/ year	Number of animals	Immunization status	Morbidity (%)	Mortality (%)	Clinical signs and symptoms
Wild goat (*Capra aegagrus*) (No. 275)	Natural reserves	Barzan	01/15; 05/15; 06/15	22	–	6 (27.3)	5 (22.7)	Paralysis of leg, fever, depression, sudden death.
		Mergasor	02/15	6	–	2 (33.3)	1 (16.7)	Depression, mucopurulent nasal discharge sudden death.
	Small zoo	Erbil	07/16	19	–	3 (15.8)	2 (10.5)	Erosive stomatitis, mucopurulent nasal discharge, conjunctivitis.
	Herd^a^	Shaqlawa	11/16	218	–	104 (47.7)	21 (9.6)	Fever, diarrhea, mucopurulent nasal discharge, congestion of conjunctiva conjunctivitis, erosive stomatitis.
	Herd	Qushtapa	17/16	10	–	3 (30)	1 (10)	Fever, diarrhea, depression, mucopurulent nasal discharge.
Domestic goat (local breed) (No. 3254)	Farm	Erbil	03/16	189	–	50 (26.5)	26 (13.8)	Fever, watery diarrhea, congestion of conjunctiva, anorexia.
		Barzan	04/16	103	–	20 (19.4)	13 (12.6)	Erosive stomatitis Paralysis of leg, fever, anorexia.
		Kalfan	08/16	208	+	74 (35.6)	17 (8.2)	Pyrexia, putrid odor to the breath, erosive stomatitis, mucopurulent nasal discharge.
		Shaqlawa	09/16	214	+	51 (23.4)	3 (1.4)	Diarrhea, anorexia, erosive stomatitis.
		Erbil	10/16	73	–	38 (52.1)	15 (20.5)	Pyrexia, mucopurulent nasal discharge, depression, pneumonia, abortion.
		Shaqlawa	12/16	233	–	107 (45.9)	31 (13.3)	Erosive stomatitis, mucopurulent nasal discharge, abortion, lameness.
		Erbil	13/16	31	–	26 (83.9)	9 (29)	Conjunctivitis, lameness, anorexia, mucopurulent nasal discharge.
		Ghoman	14/16	677	+	144 (21.3)	75 (11.7)	Pyrexia, mucopurulent nasal discharge, depression, anorexia, ocular discharge, erosive stomatitis, lameness, frothy saliva.
		Barzan	15/16	1041	–	207 (19.9)	89 (8.5)	Pyrexia, mucopurulent nasal discharge, erosive stomatitis, conjunctivitis.
		Barzan	16/16	485	–	133 (27.4)	53 (10.9)	Pyrexia, mucopurulent nasal discharge, depression, pneumonia, abortion.

^a^ = goats purchased from north of Iran and considered as transboundary animals; − = no vaccination data; + = vaccinated animals

Of the total 3254 domestic goats, PPR disease outbreak was detected with morbidity and mortality rates between 19.4 to 83.9% and 1.4 to 29%, respectively. Similarly, high morbidity (47.7%) and mortality (22.7%) rates were recorded in 275 captive wild goats (Table 1). On the other hand, the vaccinated domestic goat farms showed low mortality rate as compared to the non-vaccinated farms (Table 1). The individual farm-based and total rate of morbidity and mortality in domestic farms and wild captive goat herds are illustrated in Tables 1 and 2. Overall, the frequency of morbidity and mortality in domestic goat farms was significantly higher (*p* < 0.05) than in captive wild goat herds. Similarly, a comparative analysis on the morbidity rate was significantly different between captive wild goats located in natural reserves and three herds located in the Erbil, Shaqlawa and Qushtapa districts. In contrast, no significant difference in the mortality rate was observed. However, the rate of morbidity and mortality across Erbil city and other geographical districts (Barzan, Shaqlawa, Ghoman and Kalfan) were significantly high (*p* < 0.05) in contrast to other geographical districts where no significant difference was observed.

### Postmortem findings

Necropsy of the infected goats showed gross pathological lesions of bronchopneumonia, hemorrhages and congested lungs where trachea contained foamy fluid. Obvious hemorrhage and discontinuous streaks of congestion were observed in the mucous membranes of colon and rectum with zebra stripes lesions. Along with necrotic lesions on palatine tonsils and hemorrhage and congestion of the brain, several cases of necropsied goats showed congestion on small and large intestines with slightly or moderately enlarged mesenteric lymph nodes. Moreover, obvious hemorrhage and congestion were observed in the heart, liver, spleen and kidney (Suppl. Fig. 1A-F).

### Serological analysis

 Of the total 493 serum samples tested for the presence of PPR viral antibodies, 236 (47.9%) samples were positive. The c-ELISA results showed a high rate of sensitivity (96.2%) and specificity (91.3%). Further comparative analysis of the PPR sero-prevalence in domestic (*n* = 198; 49.7%) and captive wild (*n* = 38; 39.1%) goat serum samples showed no significant difference (Table 2).

**Table 2:** Morbidity and mortality data and detection methods (ELISA, RT-cPCR and RT-qPCR) applied for identification of PPR virus in domestic and captive wild goats

Animal	Number of herds/ farms	Number of animals	Morbidity rate (%)	Mortality rate (%)	No. of tested swabs samples/ No of positive samples (%)		
					ELISA	RT-cPCR	RT-qPCR
Domestic goats	10	3254	850 (26.1)	361 (11.1)	396/198 (50)	398/256 (64.3)	398^†^/358 (89.9)
Captive wild goats	7	275	118 (42.9)	30 (10.9)	97/38 (39.1)	88/51 (57.9)	88^§^/75 (85.2)
Total	17	3529	968 (27.4)	391 (11.1)	493/236 (47.9)	486/307 (63.2)	486/433 (89.1)

† = Total number of swab samples collected from nasal and mouth lesions (*n* = 220) and organ tissues (*n* = 178)

§ = Total number of swab samples collected from nasal and mouth lesions (*n* = 50) and organ tissues (*n* = 38)

### Phylogenetic analysis

Of the total 398 domestic and 88 captive wild goat swab samples, 256 (64.3%) and 51 (57.9%) showed positive RT-cPCR reaction for the N gene with an amplicon size of 255 bp (Table 2). Furthermore, the amino acid sequences of ten positive strains from wild (B2, C1, L81, and N11) and domestic (P49, P56, S4, V67, V89 and V90) goats were submitted to the NCBI GenBank database with the accession numbers LT629276-LT629277 and LT882721-LT882728. The sequences were further aligned and compared with the PPRV strain sequences obtained from the NCBI GenBank database.

**Fig. 2:**
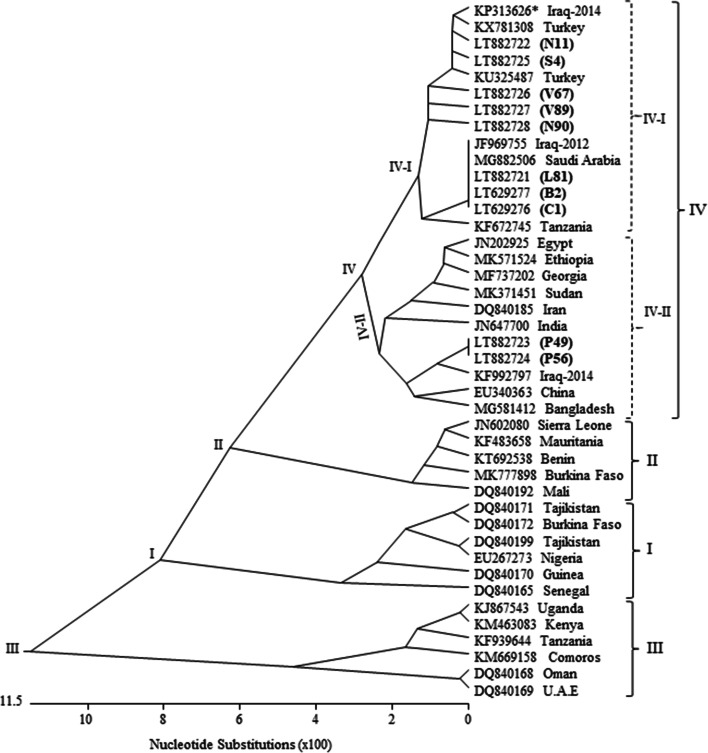
Phylogenetic tree constructed based on N gene sequences (~ 255 bp) of 10 PPRV strains (L81, N11, P49, P56, S4, V67, V89, N90, B2 and C1) showing lineages I, II, III and IV where subtype lineages I&II of lineage IV were further identified (shown as bold in parenthesis)

The phylogenic tree of ten strains and other strains of lineages I, II, III and IV revealed that the eight from wild (L81, N11, B2 and C1), and domestic (S4, V67, V89 and V90) goats strains were classified as subtype lineage IV-I. Whereas two strains (P49 and P56) from domestic goats were classified as subtype lineage IV-II (Fig. 2). The strains belong to subtype lineages I and II showed similarity between 95.3 to 97.2%. The amino acid sequences of these strains also showed similarity between 94.0 to 100% to lineage IV. Moreover, close homology for amino acid sequences of lineage I (81.0–88.1%), II (84.5–86.9%) and III (73.8–78.6%) was also observed (Suppl. Fig. 2). The amino acid sequence alignment and phylogenetic tree of these strains revealed similar results.

### RT-qPCR analysis

For RT-qPCR assay, a total of 358 (89.9%) domestic and 75 (85.7%) captive wild goat samples showed positive amplification reactions (Table 2). Moreover, of the total 270 nasal and mouth swabs collected from domestic and wild goats, 236 (87.4%) samples were positively amplified for PPRV. On the other hand, of the total 216 tissue swab samples of diseased and suspected cases, 122 (56.5%) domestic and wild goat samples were positively amplified for PPRV; however, no significant difference was detected.

## Discussion

Based on clinical investigation and laboratory identification of massive PPR disease outbreaks between 2011 and 2014, substantial loss of wild and domestic goats occurred in different geographical area in Northern Iraq. The infection re-emerged between August 2015 and September 2016 in domestic and captive wild goats in the vast geographical area that showed a high prevalence and mortality rate in the domestic goat farms as well as a high prevalence and morbidity rate in captive wild goat herds as compared to the earlier outbreaks reported in 2011 and 2014 [15, 17]. The circulation of PPRV was most likely caused by movements of infected small ruminants for trade or migration from neighboring countries. The PPRV infection was endemic in Iran and other Middle Eastern countries [18–20]. Therefore, it is worth mentioning that the PPRV infection reported in the present study occurred in the same period between 2014 to 2016 in the northern and central provinces of Iran where a high rate of mortality was reported in wild goats (*Capra aegagrus*) and sheep (*Ovis orientalis*) [20]. Information on PPR disease outbreaks between 2009 to 2019 in small ruminants with variable prevalence rates reported in different Asian countries indicates that the transmission of infection can potentially occur through cross-border migration [20–23].

The PPRV strain detected in the present study also showed a similar spectrum of clinical signs (e.g., ocular and nasal discharge, typical mouth lesions and respiratory disorders) and pathological lesions (e.g., intestinal ulceration, hemorrhage and zebra stripes lesions) [6, 15]. The reason for re-emergence of PPR infection in goats located in the Northern regions of Iraq is possibly due to the grazing of animal herds on the free range pastureland especially during the hot and dry season. Moreover, these herds move long distances in search of pasture. Also non-vaccinated herds travel long distances, which can have implications for the circulation and re-emergence of disease throughout the vast geographical area and across borders [24, 25]. The other reason for the re-emergence of infection could be the interruption or irregular mass vaccination campaigns implemented at national-level by local veterinary authorities, leading to failure of control and eradication strategies [26]. Another reason for opposing vaccination programs might be fear of vaccine side effects, which could lead to transient immunosuppression [2]. The commercial single dose of PPR vaccine used during the vaccination program contains ~ 10^3^ TCID50 of Vero cell-attenuated PPRV, which provides a strong humoral response for at least 3 years in sheep and goats. However, the vaccine needs to be stored in a cool place, and once reconstituted it must be administered to the animal within 2 h to avoid a loss of potency. Since continuous refrigeration is necessary for the entire period until the vaccine is used up, there is, therefore, an urgent need to develop a thermo-tolerant vaccine that may help in controlling and eradicating PPR disease [2, 27].

ELISA-based sero-prevalence data showed positive (47.9%) results among goats located in 17 farms and herds including three farms vaccinated with commercial PPRV live attenuated vaccine. The results are in congruence with previous sero-positive data (ranging from 38 to 70%) for PPRV in sheep and goats reported in various countries [4, 28–30]. However, a low (22.4%) and variable (between 4.7 and 34.3%) rate of antibody responses to PPRV was reported in small ruminants [7, 31]. In contrary, PPRV antibodies were not detected in domestic, wild and captive ruminants in Southern Spain [32].

Further comparative analysis between the rate of morbidity and mortality as well as rate of prevalence across different geographical locations showed a significant (*p* < 0.05) relationship, thus indicating that the PPR infection is endemic across the geographical districts. Consequently, the epidemiological prevalence of PPR virus could be similar in most regions of Northern Iraq. However, the c-ELISA results showed no significant differences between domestic goat farms and captive wild goat herds, which could possibly be due to an uneven distribution of the respective animals investigated. The c-ELISA results suggest that there is a contagious nature of infection through the transmission of PPRV from infected to susceptible animals. Moreover, high prevalence of PPRV antibodies could be due to animals that were recovered from infections or they were in the clinical stage or animals that had been previously vaccinated. Therefore, movement of infected or sub-clinically infected animals is one of the most important factors that plays an important role in the transmission of PPRV to healthy animals in the geographical area.

Based on the phylogenetic tree, the sequences showed a close relation to strains detected in 2012 and 2014 [15, 17]. The epidemiological form of PPR infection, first confirmed in Northern Iraq in 2011 revealed type IV lineage [15]. However, eight and two strains detected in the present study were classified to subtype lineage IV-I and IV-II, respectively. Lineage IV was associated with previous outbreaks reported from neighboring Asian countries [22, 33, 34].

These study results indicate that lineage IV has been persistent over recent years, and there is a complex epidemiological prevalence of PPR disease in the region. The phylogenetic analysis suggests recent co-circulation of lineage IV between these neighboring nations. However, two subtype lineages were identified within lineage IV where subtype lineage IV-I was identified in domestic and wild goats, and subtype lineage IV-II only detected in domestic goats suggesting an independent introduction thereof followed by evolution of the virus. While serological data of PPR viral infection has been reported in the region, to our knowledge, this is the first report on the detection of lineage IV subtype lineages I and II in the region suggesting that PPRV lineage IV possibly re-emerged and sub-lineages were re-introduced in Iraq. Since information on domestic and wild goat stock kept is neither clear nor reliable, therefore, the origin of novel subtype lineages could not be determined. Further surveillance and analyses are necessary to characterize the impact of these novel subtype lineages in other neighboring countries. In order to minimize the circulation of PPR virus, there is an urgent need to establish quarantine check points at the borders. This would help in tracking these virus strains more accurately. Although this is a difficult task, it can possibly be achieved by cooperation from goat suppliers and between countries in the region with the goal to develop and implement regulations to control transboundary spread of PPR disease. Moreover, regular monitoring of known and novel strains of PPRV will be important for implementation of control measures and selection of appropriate vaccine strains. These actions will substantially aid in minimizing the prevalence of disease and reducing the socio-economic burden, improving animal health and reproduction as well as sustainable growth of livestock industry. These measures will also help to enhance farm management practices by establishing a PPR virus eradication program including a regular monitoring and vaccination program to mitigate the risk of re-emergence of PPR virus infection in domestic and captive wild goats in Iraq.

## Conclusions

This study provides information on the re-emergence of PPR virus infection detected and characterized in domestic and captive wild goats in various regions of Iraq where two novel subtypes (I and II) of lineage IV were identified. The outbreak had comparatively high mortality in captive wild goat herds than domestic goat farms as compared to morbidity. However, a high frequency for anti-PPRV nucleoprotein antibodies and N gene were detected in both domestic and wild goats. The data based on clinical, pathological, serological and genotypical analyses would contribute to tracking the PPRV spread as well as improving on-site control and trade regulation strategies. The animal movement regulations between Iraq and neighboring countries have not been strictly followed which facilitates PPRV circulation within the region. Additionally, the presence of novel subtype lineages in domestic and captive wild goats suggests that the transmission of this virus can potentially occur within the region.

## Methods

### Study area description and clinical observations

**Fig. 3:**
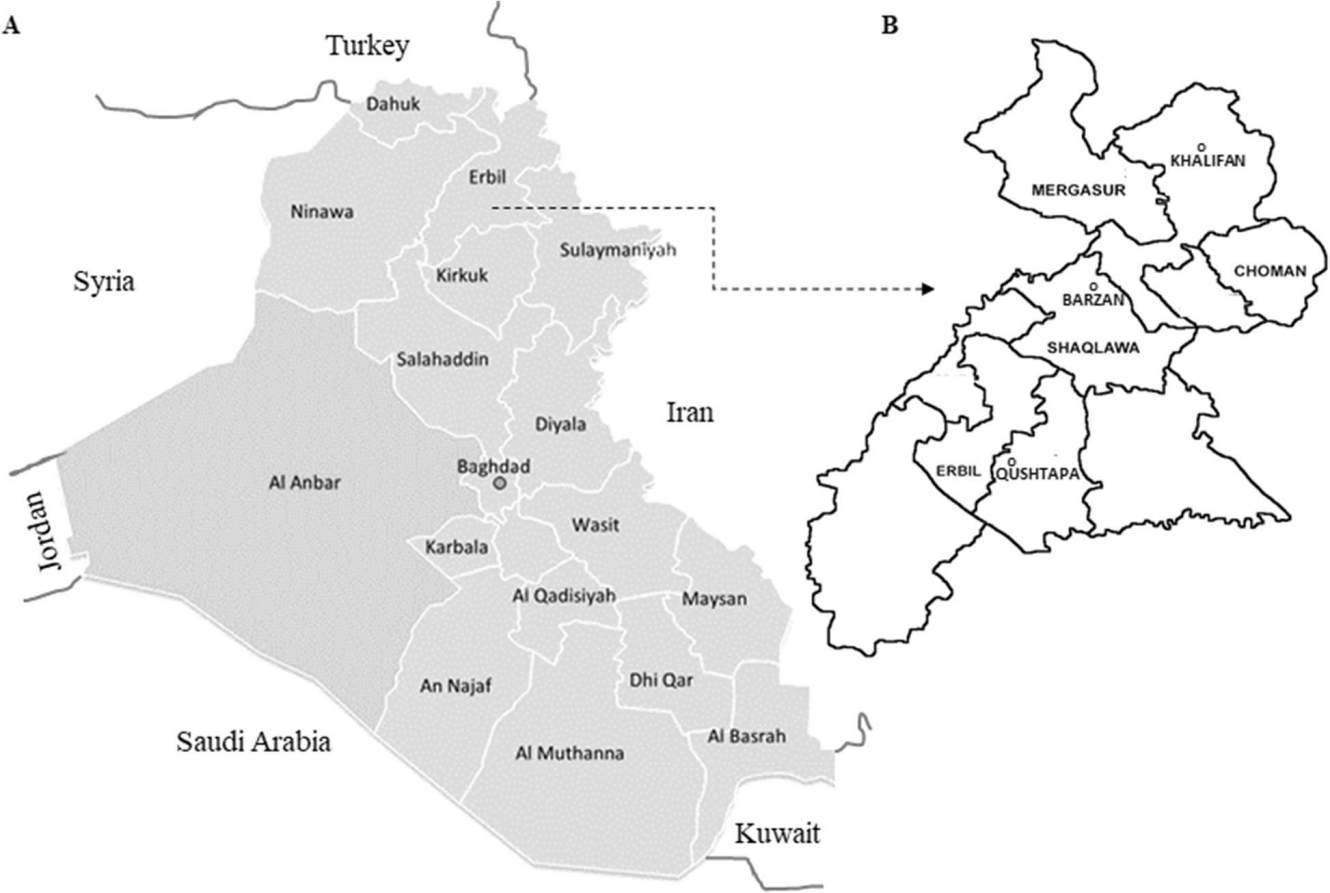
**A** Map of Iraq showing Erbil Governorate, part of Kurdistan region, located in Northern Iraq. **B** Map of districts of the Erbil Governorate and seven geographical [Erbil (provincial capital), Mergasur, Khalifan, Choman, Barzan, Shaqlawa and Qushtapa] locations along with coordinates investigated in this study. In addition, two natural reserves located in the mountains of Mergasur and Barzan regions are marked

The investigation was conducted in seven geographical districts including Mergasur, Khalifan, Choman, Barzan, Shaqlawa, the provincial capital Erbil city and Qushtapa located in Erbil Governorate, in the Kurdistan region, Northern Iraq (Fig. 3A&B). Erbil Governorate (13.165 km^2^) is adjacent to the international borders of Southern Turkey and Northwestern Iran characterized by a rugged mountain range along the northern border of Iraq where the Mergasur region and Shaqlawa mountains (Barzan) are natural reserves for rearing wild goats (bezoar ibex, *Capra aegagrus*).

A total of 3529 goats including 3254 domestic goats (local breeds) from ten farms located in five geographical areas (Barzan, Ghoman, Kalfan, Erbil city, Shaqlawa) as well as 275 captive wild goats from seven herds located in five geographical areas (Barzan, Mergasor, Erbil, Shaqlawa, Qushtapa) were studied. A total of seven herds of captive wild goats including four herds reared by free-range grazing in two natural reserves in the mountainous area of Mergasur district and Brazan city, one herd reared in a small animal zoo located in Erbil city, as well as two commercial herds, located in Erbil city and Shaqlawa district were investigated (Table 1). The latter farm transported wild captive goats from mountainous regions of Northern Iran to Northern Iraq for breeding purpose. All farms and herds were visited by local veterinary authorities from August 2015 to September 2016 for clinical examination, data and sample collection. The domestic goat farms were managed and reared in a semi-free range grazing system where the goats spent most of their time (~ 60–70%) grazing on free local rangeland. Of the total ten domestic goat farms and seven captive goat herds, 13 were not immunized against PPR virus while three domestic goat farms were vaccinated with commercial dried live attenuated vaccine strain Nig. 75/1 (Jordan Bio-Industries Center, Amman, Jordan) (Table 1).

### Clinical and post-mortem examination and observations

The veterinarians recorded the health status including the form of disease (peracute, acute and subacute) and case history of any symptoms related to PPR infection including pyrexia, ocular and nasal discharge, ulceration and erosion of oral mucosa (stomatitis), emaciation, severity of diarrhea as well as morbidity and mortality status. The infected domestic goats were euthanized using pentobarbital (1 mL per 10 lb. (4.5 kg) body weight) via intravascular injection. The post-mortem investigation was conducted by collecting necropsy samples from euthanized and fresh carcasses at the Directorate of Central Veterinary Laboratory (DCVL), Erbil, Iraq.

### c-ELISA

For preparation of serum for c-ELISA, a total of 493 (domestic: *n* = 396; wild: *n* = 97) goat blood samples were collected and placed on ice in a cooler and transported to the DCVL, Erbil, Iraq. The serum samples were tested for antibodies against PPR virus using commercial ELISA kit (ID Screen®PPR Competition, Innovative Diagnostics, Grabels, France) and BioTek Absorbance Microplate reader 800 TS (BioTek Instruments GmbH, Bad Friedrichshall, Germany) for detecting anti-PPRV nucleoprotein antibodies in sheep and goat serum. The optical density (OD) was recorded at 450 nm in accordance with the manufacturer’s recommended procedure. The results were recorded as a percent inhibition of the optical density (%OD) reading of the test sample, and the competition percentage (S/N%) for each sample was calculated using following formula: S/N% = OD_sample_/OD_NC_ × 100. Sample results ≤OD %50 and > OD%60 were considered as positive and negative, respectively. The serological results, based on the proportion of animals that had detectable antibodies in the sample population, were calculated and recorded as a percentage.

### Genotypic identification and characterization of PPRV

#### Sample collection

A total of 486 swabs samples including 270 (*n* = 220 domestic goats; *n* = 50 wild goats) swabs from nasal and mouth lesions and 216 (*n* = 178 domestic goats; *n* = 38 wild goats) swabs from tissue samples were collected from different organs including lymph nodes, small and large intestine, lung and liver of clinically diseased and suspected healthy animals. All samples were placed on ice in a cooler and delivered to the DCVL, Molecular Department for further investigation.

#### RNA extraction and complementary DNA synthesis

RNA was extracted from nasal and mouth swabs as well as from organ tissues using RNeasy Mini Kit (Qiagen GmbH, Hilden, Germany) according to the manufacturer’s instruction. Synthesis of cDNA was carried out in a 25 μL reaction where 3.5 μL of the purified RNA was added to a mixture containing 2 μL One *Taq* One-Step Enzyme, 2.5 μL buffer (New England BioLabs Inc., Ipswich, MA, USA), 2 μL reverse specific forward primer NP3, 4 μL 10 mM dNTP, 1.0 μL MgCl_2_ and 11 μL Aqua dest using One *Taq*® One-Step RT-PCR kit (New England BioLabs Inc.). The reaction was carried out at 42 °C for 60 min (one cycle) and 95 °C for 10 min (one cycle) using Eppendorf MasterCycler Gradient PCR system (Eppendorf AG, Hamburg, Germany). The purified complementary DNA **(**cDNA) was quantified using NanoDrop 2000 (Thermo Fisher Scientific GmbH, Darmstadt, Germany) and stored at − 20 °C for further analysis.

#### Reverse transcription complementary polymerase chain reaction (RT- cPCR)

The cDNA was investigated for the nucleocapsid protein (N) gene using Goldstar PCR Red Master mix (Eurogenetec, Deutschland GmbH, Köln, Germany), oligonucleotide forward primer NP and reverse primer NP4 (Eurofins Genomics GmbH, Ebersberg, Germany) as previously described [35]. The PCR amplification was performed in a 30 μL reaction mixture with 1 μL (10 pmol μL^− 1^) of each primer, 15 μL ready-to-use Goldstar PCR Red Master mix and 10 μL Aqua dest. Finally, 3 μL DNA template was added to each reaction tube. The amplification reaction was carried out with the thermocycler program: Initial denaturation at 94 °C for 2 min followed by 35 cycles consisted of 94 °C for 30 s, 55 °C for 60 s, 72 °C for 30 s and final extension 72 °C for 5 min. The amplified PCR products were electrophoresed on 1.5% agarose gel matrix (Biozym Scientific GmbH, Hessisch-Oldendorf, Germany), stained in ethidium bromide (0.5 μg mL^− 1^) and visualized at 302 nm on a UV transilluminator (Cleaver Scientific Ltd., Warwickshire, UK).

#### PCR-based sequencing and phylogenetic analysis

The positive PCR amplicons of ten randomly selected samples obtained from domestic and wild goats were further sequenced to gain information about the individual N gene structure. PCR products were purified using QIAquick PCR purification kit (Qiagen, GmbH) according to the manufacturer’s instructions. Quality and concentration of purified PCR products were confirmed as mentioned in the above section. The purified PCR products were sequenced at Seqlab-Sequence Laboratories GmbH (Göttingen, Germany), and the sequences were analyzed using FinchTV (version, 1.4.0). For confirming sequence identity, sequence data was subjected to Nucleotide BLAST search (https://blast.ncbi.nlm.nih.gov/Blast.cgi) against the global database. Additionally, all sequences were further analyzed and compared by Clustal-V and Clustal-W pairwise multiple sequence alignment using MegAlign (DNASTAR Inc., Madison, WI, USA). The phylogenetic tree was constructed where bootstrapping was performed by creating 1000 trials. Similarly, the nucleotide sequences were converted to amino acid sequences using the translate DNA step of the EditSeq program (DNASTAR Inc.) and the phylogenetic tree was constructed using the MegAlign program.

#### Reverse transcription quantitative PCR (RT-qPCR)

For the quantitative detection of the specific target gene of the PPR virus, cDNA samples were investigated for the N gene using the VetMAX™ PPRV reagents containing N gene-specific oligonucleotide primers and TaqMan® real-time PCR master mix in accordance to the manufacturer’s instructions (Thermo Fisher Scientific GmbH, Dreieich, Germany). All samples were tested along with external (inactivated bacteria or virus) and internal (exogenous) positive controls (Thermo Fisher Scientific GmbH). The qPCR assay was performed using 7500 real-time PCR (Applied Biosystems GmbH, Darmstadt, Germany) system where each sample was analyzed in duplicate. The samples were considered positive when the cycle threshold (Ct) value was ≤40.0 and ≥ 41 Ct value was considered as negative or indeterminate. The quality and reaction efficiency were calculated based on Ct values whereas correlation coefficient values were obtained from the standard curve.

### Data analysis

To compare and assess the geographical difference and rate of morbidity and mortality between domestic goat farms and captive wild goat herds, McNemar Chi-square Contingency and Fisher’s Exact Tests were applied using ELISA and RT q-PCR-based data for identifying significant difference (*p* < 0.05).

## Supplementary Information

**Additional file 1: Supplementary Fig. 1.** Clinical symptoms of PPR infection observed in domestic and captive wild goats. Panels A-F: A: Mucopurulent nasal discharge; B: Hyperemia of conjunctival tissue (conjunctivitis); C&D: Ulcers and erosive stomatitis; E: Diarrhea; F: Sudden death of wild goat.

**Additional file 2: Supplementary Fig. 2.** Alignment of N gene amino acid sequences (84 amino acid = ~ 255 bp) of PPRV strains (L81, N11, P49, P56, S4, V67, V89, N90, B2 and C1) (shown as bold) identified in the present study and amino acid sequences of lineage I, II, III and IV obtained from NCBI Genbank database. Differences from consensus depicted in marked letters where blank spaces (−) denotes lack of amino acid sequences.

## Data Availability

The data generated and analyzed in this study are available upon request. Additionally, the sequence datasets generated and analyzed are available in the NCBI GenBank database with the accession numbers LT629276-LT629277 and LT882721-LT882728.

## Abbreviations

PPRV: Peste des Petits Ruminants Virus
DCVL: Directorate of Central Veterinary Laboratory
c-ELISA: competitive enzyme-linked immuno-sorbent
b-ELISA: blocking enzyme-linked immuno-sorbent
RT-qPCR: Real-time quantitative PCR
RT-LAMP: Reverse transcription loop-mediated isothermal amplification
cDNA: complementary DNA
