# Supplementary figures and images for: Pheno- and genotypic characterization and identification of novel subtypes of Peste des Petits Ruminants virus in domestic and captive wild goats in Northern Iraq
Source: BMC Microbiol. 2021 Dec 7;21:334. doi: 10.1186/s12866-021-02372-2 (PMC8650381; doi:10.1186/s12866-021-02372-2)

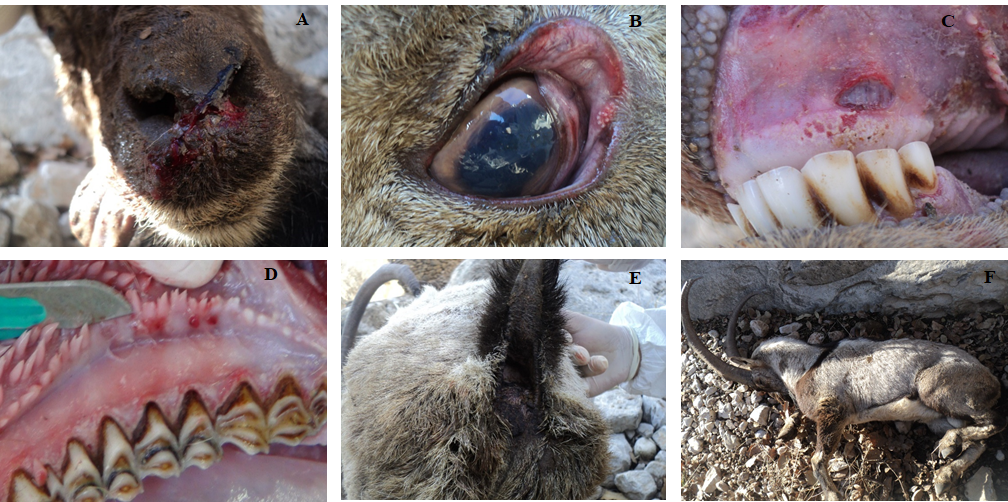

Supplement: Supplementary file 1 — Additional file 1: Supplementary Fig. 1. Clinical symptoms of PPR infection observed in domestic and captive wild goats. Panels A-F: A: Mucopurulent nasal discharge; B: Hyperemia of conjunctival tissue (conjunctivitis); C&D: Ulcers and erosive stomatitis; E: Diarrhea; F: Sudden death of wild goat. [file 12866_2021_2372_MOESM1_ESM.tif]

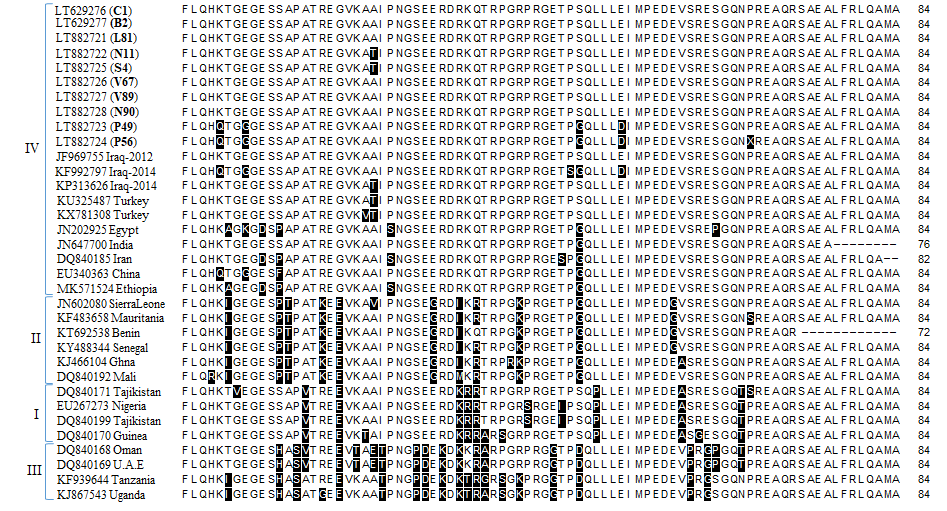

Supplement: Supplementary file 2 — Additional file 2: Supplementary Fig. 2. Alignment of N gene amino acid sequences (84 amino acid = ~ 255 bp) of PPRV strains (L81, N11, P49, P56, S4, V67, V89, N90, B2 and C1) (shown as bold) identified in the present study and amino acid sequences of lineage I, II, III and IV obtained from NCBI Genbank database. Differences from consensus depicted in marked letters where blank spaces (−) denotes lack of amino acid sequences. [file 12866_2021_2372_MOESM2_ESM.tif]
